# Therapeutic Potential of Mesenchymal Stem Cells (MSCs) and MSC-Derived Extracellular Vesicles for the Treatment of Spinal Cord Injury

**DOI:** 10.3390/ijms222413672

**Published:** 2021-12-20

**Authors:** Gang-Un Kim, Soo-Eun Sung, Kyung-Ku Kang, Joo-Hee Choi, Sijoon Lee, Minkyoung Sung, Seung Yun Yang, Seul-Ki Kim, Young In Kim, Ju-Hyeon Lim, Min-Soo Seo, Gun Woo Lee

**Affiliations:** 1 Department of Orthopedic Surgery, Hanil General Hospital, 308 Uicheon-ro, Dobong-gu, Seoul 01450, Korea; yume14os@gmail.com; 2 Department of Laboratory Animal Center, Daegu-Gyeongbuk Medical Innovation Foundation (DGMIF), Daegu 41061, Korea; sesung@dgmif.re.kr (S.-E.S.); kangkk@dgmif.re.kr (K.-K.K.); cjh522@dgmif.re.kr (J.-H.C.); sjlee1013@dgmif.re.kr (S.L.); tjdalsrud27@dgmif.re.kr (M.S.); 3 Department of Biomaterials Science, Life and Industry Convergence Institute, Pusan National University, Miryang 50463, Korea; syang@pusan.ac.kr; 4 Efficacy Evaluation Team, Food Science R&D Center, KolmarBNH CO., LTD, 61Heolleungro 8-gil, Seocho-gu, Seoul 06800, Korea; lovesshot@kolmarbnh.co.kr; 5 Cellexobio, Co. Ltd., Daegu 42415, Korea; kimyoungin@cellexobio.com; 6 New Drug Development Center, Osong Medical Innovation Foundation, Chungbuk 28160, Korea; globaljh2019@gmail.com; 7 Department of Orthopedic Surgery, Yeungnam University College of Medicine, Yeungnam University Medical Center, 170 Hyonchung-ro, Namgu, Daegu 42415, Korea

**Keywords:** mesenchymal stem cell, extracellular vesicle, exosome, spinal cord injury

## Abstract

Spinal cord injury (SCI) is a life-threatening condition that leads to permanent disability with partial or complete loss of motor, sensory, and autonomic functions. SCI is usually caused by initial mechanical insult, followed by a cascade of several neuroinflammation and structural changes. For ameliorating the neuroinflammatory cascades, MSC has been regarded as a therapeutic agent. The animal SCI research has demonstrated that MSC can be a valuable therapeutic agent with several growth factors and cytokines that may induce anti-inflammatory and regenerative effects. However, the therapeutic efficacy of MSCs in animal SCI models is inconsistent, and the optimal method of MSCs remains debatable. Moreover, there are several limitations to developing these therapeutic agents for humans. Therefore, identifying novel agents for regenerative medicine is necessary. Extracellular vesicles are a novel source for regenerative medicine; they possess nucleic acids, functional proteins, and bioactive lipids and perform various functions, including damaged tissue repair, immune response regulation, and reduction of inflammation. MSC-derived exosomes have advantages over MSCs, including small dimensions, low immunogenicity, and no need for additional procedures for culture expansion or delivery. Certain studies have demonstrated that MSC-derived extracellular vesicles (EVs), including exosomes, exhibit outstanding chondroprotective and anti-inflammatory effects. Therefore, we reviewed the principles and patho-mechanisms and summarized the research outcomes of MSCs and MSC-derived EVs for SCI, reported to date.

## 1. Introduction

Spinal cord injury (SCI) is a life-threatening, devastating injury to the spinal cord, leading to temporary or permanent changes to the cord, accompanied by partial or complete loss of motor, sensory, and autonomic functions [1,2]. SCI frequently leads to paralysis called paraplegia or quadriplegia, with sensory dysfunction below the injury level [3]. It generally induces severe psychological and economic burdens on patients and healthcare systems [4,5], and can negatively affect the majority of basic bodily functions, such as breathing, bowel and bladder function, hormone release, and sexual function, because of the loss of connection between the brain and the peripheral nervous system [6]. It is estimated that the prevalence and incidence of SCI worldwide is 236–4187 per million people, with up to 770,000 new cases per year and is more common in males below 30 years of age [7,8,9].

Initial mechanical insult caused by physical forces such as contusion, compression, transection, or stretching of the spinal column generally causes spinal cord disruption and results in primary injury [10,11,12,13,14]. Primary injury is defined as immediate mechanical injury to the spinal cord, which is an irreversible process [15,16]. A primary injury is followed by a cascade of secondary injuries, exacerbating the condition of the injured spinal cord [17,18]. Secondary injury occurs within minutes of the primary mechanical injury, manifested as local vascular damage, subsequent progressive hemorrhage, and ischemia, edema, thrombosis, ionic changes, oxidative stress through the release of free radicals, lipid peroxidation, excitotoxicity, and cell death promoted by apoptosis and cell necrosis [2]. Furthermore, the inflammatory reaction and gliosis hyperplasia, following the formation of an inhibitory environment and scar formation, interfere with axonal regeneration and limit the therapeutic potential [19,20].

Despite recent clinical advances in SCI management showing some improvement in patients’ quality of life, recovery from SCI remains substantially limited [21,22]. Pathologic targets for the treatment of SCI can be divided into three broad categories. First, surgical decompression and the removal of mechanical compressing material of the spinal cord at the initial trauma [2,23,24,25,26,27]. Second, anti-inflammatory treatments for the level around the injured spinal cord. There are some considerable inflammatory and anti-inflammatory mechanisms and factors, from the initial spinal cord injury to the late chronic phase [28,29,30]. Third, axonal regeneration at the site of the spinal cord injury, set as the ultimate goal for the treatment of spinal cord injuries [6]. With the initial SCI, macrophages are intensely infiltrated into the damaged lesion, and contribute to form a cavity of injury (COI) around the injured site, which cut off the neuronal regeneration [31,32,33,34]. Axonal regeneration is disturbed by scar [35] and the COI lesions that filled with fluid, because the axons do not have an ability to cross the liquid content in the COI without any bridge-like structures to cross it [36]. In addition, arachnoiditis, a granulomatous infiltration around the damaged spinal cord, contributes to the formation of a mature scar that does not contain astrocytes or other glial cells [20]. The prognosis of SCI patients remains abysmal, the mortality rate remains high, and life expectancy is significantly shortened [37].

Stem cell transplantation therapy for damaged spinal cords is a promising therapeutic strategy for replacing the damaged neuronal cells and creating an environment conducive to repair [3]. Cell therapies show neuroprotective and regenerative potential in SCI with diverse targets and stimulative responses, including regulation of inflammatory reactions, nutritional support, and promotion of neuronal plasticity [38]. Several types of stem cells have been tested, or are currently being tested, clinically for SCI treatment [39]. The majority of the experimental and clinical trials to treat SCI used mesenchymal stem cells (MSCs) isolated from bone marrow (BM-MSCs), umbilical cord MSCs (U-MSCs), and adipose tissue MSCs (AD-MSCs). Known mechanisms of MSCs to treat SCI include suppression of inflammation to limit secondary injury, secretion of paracrine factors that protect the remaining axons and promote axonal regeneration, and differentiation of MSCs into nerve cells to replace the damaged nerve cells [40,41]. MSC synthesis of neurotrophic and angiogenic factors promotes neuronal survival and regeneration. Furthermore, high biosafety and immunomodulation of MSCs make them the most promising cell type for SCI regenerative therapy [42]. However, despite these promising results of MSCs in SCI therapy, certain studies have reported that MSCs have numerous drawbacks and that their therapeutic properties are more likely to be due to their paracrine action [43].

In this review, we introduce the cutting-edge status of SCI treatment with MSCs and MSC-derived EVs, focusing on the potential therapeutic mechanisms with experimental and clinical trial results. Furthermore, we discuss the prospects, current limitations, and challenges of MSCs and MSC-derived EVs, along with hopes for future promising therapeutic methods for SCI.

## 2. Pathophysiology of Spinal Cord Injury

**Figure 1:**
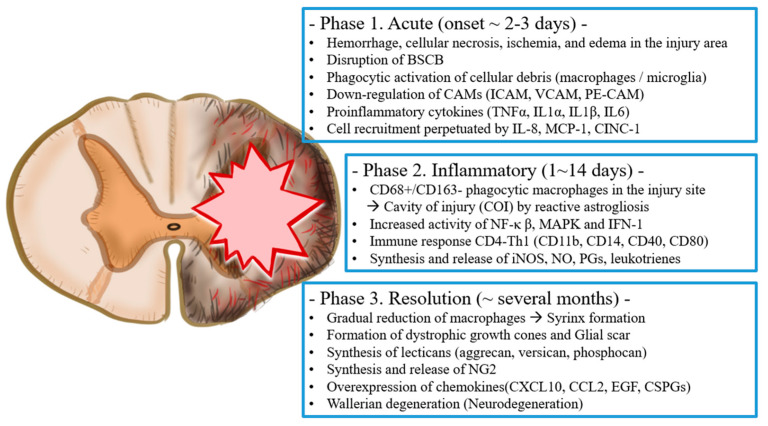
Schematic diagram for damage stages and responses in spinal cord injury.

The pathological process of SCI can be divided into two consecutive phases: primary and secondary injury [44,45]. Primary injury occurs immediately at the time of injury, and secondary injury begins within minutes of the primary injury [46] (Figure 1).

Primary injury is an immediate mechanical injury caused by physical force that causes irreversible damage to the spinal cord [10,11,15,16]. The initial mechanical force leads to the rupture of the axonal membranes of the spinal cord and the release of inhibitory materials from the myelin sheath, including neurite outgrowth inhibitor protein A, myelin-associated glycoprotein, oligodendrocyte myelin glycoprotein, and chondroitin sulfate proteoglycan, all of which are powerful inhibitory factors for axonal regeneration [47,48,49,50,51,52].

Secondary injury is delayed and progressive, presenting within minutes of mechanical insult. In addition to mechanical damage, such as cord hemorrhage, swelling, ischemia, and blood-spinal cord barrier (BSCB) disruption, inflammatory cells release inflammatory cytokines due to BSCB destruction [53,54,55]. Secondary injury negatively affects cell survival in the damaged neuronal tissues and also the surrounding tissue, causing an enlargement of the lesion into the adjacent spinal cord segments in rostro-caudal directions [13]. Secondary injury includes electrolyte shifts, free radical generation, and release of toxic compounds and excitatory amino acids that trigger cell necrosis and apoptosis at the injured site [56,57,58,59,60,61,62,63,64,65]. Furthermore, proinflammatory cytokines and chemokines, such as interleukin (IL)-1β, IL-6, and tumor necrosis factor (TNF)-α, promote the differentiation of neural stem/progenitor cells into astroglia, resulting in the formation of scar tissue [66,67,68]. Tissue necrosis and cavity and scar formation, combined with axonal degeneration, ultimately impede functional recovery [69,70]. In the early phase of secondary injury, the glial scar plays a positive role in the injury site through BSCB regeneration and by limiting inflammation and toxic compounds and removing debris. However, at the later phase of injury, glial and fibrotic scars, along with axonal growth inhibitors, interfere with neuronal regeneration [71,72].

The BSCB plays a role in maintaining the normal function of the nervous system, and its unique properties and functions are regulated by neurovascular unit cells [73]. The BSCB is composed of the basement membrane, pericytes, capillary endothelial cells, and astrocyte foot processes [74]. The blood vessels at the site of injury are destroyed immediately after SCI, and the BSCB far from the injury area is permanently destroyed [75]. Loss of barrier integrity leads to increased permeability and the inflow of toxic materials into the injured spinal cord, resulting in edema and death of neuronal cells [76]. Therefore, ensuring that the integrity of the BSCB is uncompromised might be a potential target for SCI treatment. Pericytes, as a part of the neurovascular unit, are essential for the formation, maintenance of integrity, and function of the microvessels and BSCB. Pericytes have the ability to secure the stability of microvessels via three possible mechanisms: promoting the expression of endothelial tight junction proteins, regulating vesicle transport and body flow across cells, and moderating the tightness connection arrangement [77].

Neuroinflammation is characterized by the activation of local resident immune cells, and this activation is arbitrated by a protein complex-inflammasome called the nucleotide-binding domain-like receptor protein 3 (NLRP3) inflammasome. This inflammasome plays a very important role in SCI secondary injuries [78]. The NLRP3 inflammasome is located in the cytoplasm and is involved in the regulation of natural immune reactions [79,80]. Animal experimental models have shown that the NLRP3 inflammasome may be triggered and up-regulated following SCI and that inhibition of the NLRP3 inflammasome promotes functional recovery after SCI [81,82,83,84,85].

In addition, both classic and alternative complement pathways in the local immune response can be activated after SCI [86]. Activation of these pathways may exacerbate inflammatory reactions in the secondary injury process. Complements C1q and C3 are known to be related to the NF-κB signaling pathway [87,88], and secondary injury in SCI is regulated by NF-κB [84]. Thus, inhibiting NF-κB may be a possible mechanism for minimizing the inflammatory reaction and promoting functional recovery after SCI.

Macrophages are also involved in immune regulation in SCI secondary injuries. The polarization of macrophages determines their role in the inflammatory process [89]. The CD68^+^ phenotype is known as the pro-inflammatory macrophage, induced by TNF-α, IFN-γ, and IL-6 [89], while the CD163^+^ phenotype, known as the anti-inflammatory macrophage, is induced by IL-10. At the initial SCI site, myelin damage induces the infiltration of numerous macrophages into the site of necrosis via chemotaxis. The prevalence of CD68^+^/CD163^-^ macrophages, which are the pro-inflammatory phenotypes around the COI, exhibit the severe inflammation and further contribute to progressive spinal cord destruction through beyond 16 weeks from the initial SCI [20,90]. Persistence of CD68^+^/CD163^-^ macrophages showed an ongoing severe inflammatory state of the SCI lesion, and the gradual decline of them indicates a progressive increase in the anti-inflammatory process.

Astrocytes have the ability to hinder or promote recovery of the central nervous system (CNS); thus, they play a very important role in the SCI process [91,92,93,94,95]. Two phenotypes of reactive astrocytes, A1 and A2 astrocytes, pre-present and are induced by neuroinflammation and ischemia. A1 astrocytes, generally formed immediately after SCI induced by IL-1α, TNF-α, and C1q, have neurotoxic effects on myelin, synapses, and neurons that can lead to neuronal and oligodendrocyte death [96]. Contrarily, A2 astrocytes exert a protective function by up-regulating the expression of certain neurotrophic factors [91]. Therefore, selective inhibition of A1 astrocytes may be a potential SCI treatment strategy. Furthermore, it has recently been shown that reactive astrocytes eliminate red blood cells (RBCs) around SCI lesions through phagocytosis. This mechanism, named the astrocytic erythrophagocytosis, is considered to contribute to the rapid removal of scattered RBCs around the injured site to prevent macrophage aggregation and associated destructive inflammation.

Recently, microRNAs (miRNAs) have been shown to be involved in tissue injury and regenerative processes, and several miRNAs have attracted attention as potential targets for SCI treatment. miRNAs are endogenous non-coding RNAs with a length of 20–24 nucleotides that cause translational inhibition and degradation of these target messenger RNAs (mRNAs) [97,98]. miRNA-21 expression increases in the injury of various tissues and organs. It reduces neuronal apoptosis by promoting the activation of the phosphatase and tensin homolog-protein kinase B (Akt) signaling pathway [99] and regulating the expression of apoptosis-related proteins such as Bax, Bcl-2, caspase-9, and caspase-3 [100,101]. miRNA-133b also plays a key role in neuronal differentiation, growth, and apoptosis [102,103,104]. Yu et al. showed that reduced miRNA-133b expression reduces neuronal axonal regeneration and does not help recover motor function [105]. It has recently been shown that miRNA-126 promotes functional recovery after SCI. Hu et al. reported that miRNA-126 expression decreases after SCI, whereas increasing miRNA-126 levels appear to reduce inflammation and promote angiogenesis and functional recovery [106].

## 3. Mesenchymal Stem Cells for the Potential Treatment of Spinal Cord Injury

Stem cell transplantation therapies in SCI show neuroprotective and regenerative potential with different targets and responses [38]. Among the various stem cells currently available, certain inherent properties of MSCs are advantageous over other stem cells in the research; besides, MSCs are easier to harvest and isolate, and they have low immunogenicity [107,108,109]. Moreover, MSCs have fewer ethical considerations than other types of stem cells, such as embryonic stem cells [110]. The core capabilities of MSCs, such as homing, proliferation, differentiation, secretion, and immunomodulatory abilities, are of considerable interest for SCI treatment [111]. The International Society for Cellular Therapy position statement defined MSCs as cells that (1) adhere to plastic in culture conditions; (2) express CD105, CD73, and CD90, but not CD45, CD34, CD14, CD11b, CD79alpha, CD19, and HLA-DR surface molecules; and (3) are able to differentiate into osteoblasts, adipocytes, and chondroblasts in vitro [112].

MSCs are also known to have the ability to differentiate into neural cells and express neuronal markers [113,114,115,116] through specific procedures. Traditionally, the regenerative potential of MSCs is thought to be due to cell plasticity [117,118]. Although MSCs have the ability to differentiate into various neural and glial cells, most of their effects are based on their paracrine action [119]. MSCs produce and release a broad range of bioactive molecules, called secretomes. Proteomic analysis of secretomes revealed that they contain trophic factors and cytokines, such as growth factors, immunomodulators, and antioxidants [120]. Therefore, paracrine factors from MSCs have diverse functions, including anti-inflammatory, anti-apoptotic, extracellular matrix modulatory, and neuroprotective actions, by protective action against fibrosis, apoptosis, and oxidative damage [121].

Certain molecules, including vascular endothelial growth factor (VEGF), hepatocyte growth factor (HGF), insulin-like growth factor-1 (IGF-1), stanniocalcin-1, transforming growth factor-β (TGF-β), and granulocyte-macrophage colony-stimulating factor, promote the survival of damaged neurons and oligodendrocytes [122,123]. They also stimulate angiogenesis along with placental growth factor, monocyte chemoattractant protein-1, basic fibroblast growth factor (bFGF), and IL-6 [124]. Stimulation of neural cell proliferation and regeneration is mediated by glial cell-derived neurotrophic factor, brain-derived neurotrophic factor (BDNF), and nerve growth factor (NGF) [125]. MSCs express their immunomodulatory actions through cell-to-cell contact and the secretion of IL-10, TGF-β, PGE-2, galectin-1, indolamine 2,3 dioxygenase (IDO), and HLA-G [122,126,127,128].

By modulating inflammation, MSCs reduce neural damage to the remaining and surrounding unaffected tissues from secondary injury. MSCs can also improve neurite growth by improving the environment of the extracellular matrix by inhibiting gliosis [129]. In addition, antioxidant properties, direct cell fusion, mitochondrial transfer, and production of MSC microvesicles have been reported to exert their therapeutic effect [130,131,132].

MSCs are available from different tissues, including BM-MSCs, U-MSCs, AD-MSCs, neural stem cells, neural progenitor cells, embryonic stem cells, and induced pluripotent stem cells [38]. Among them, BM-MSCs, U-MSCs, and AD-MSCs, which have undergone the most studies and clinical trials, will be described in more detail in the subsequent sections.

### 3.1. Bone Marrow Mesenchymal Stem Cells

The bone marrow is the most popular source of MSCs. BM-MSCs are partially differentiated progenitor cells present in adult bone marrow. They are considered pluripotent, capable of differentiating into neurons and glial cells, and are involved in continuous hematopoiesis and bone regeneration [133,134]. However, further studies revealed that BM-MSC therapy is mainly involved in cell fusion and transdifferentiation, instead of cell differentiation. The introduction of BM-MSCs to the injury site showed a beneficial role in recovery from SCI by reducing the inflammatory reactions and astroglial scarring density [135,136], improving the microenvironment of the injury site, enhancing the nutritional support, and reducing the BSCB leakage [137]. Therefore, BM-MSCs might have diverse treatment potential for SCI because of their reduced immunogenicity and improved availability [38,138]. Interestingly, these beneficial effects occurred similarly when BM-MSCs were administered locally into the spinal cord cavity [139], intrathecally [140], or systemically [141,142]. In an animal experimental model of the BM-MSC intravenous graft, functional recovery of SCI was achieved through the expansion of neurotrophic factors, including NGF, BDNF, and VEGF, which are key regulators of neuronal differentiation, initiation, and maintenance of angiogenesis [142,143,144].

Jeon et al. performed a phase I trial in which BM-MSCs were administered into the intramedullary space (8 × 10^6^ cells) and intradural space (4 × 10^7^ cells) in 10 patients with SCI. Long-term follow-up of the patients showed that three patients with American Spinal Injury Association (ASIA) impairment scale (AIS) grade B improved their motor power of the upper extremities with better activities of daily living [145]. Furthermore, Saito et al. confirmed that significant improvement was observed in two patients with AIS grades B and C following BM-MSC therapy [146]. El-Kheir et al. reported an improvement in AIS grade in 17 out of 50 BM-MSC-treated patients combined with physiotherapy [147]. Karamouzine et al. administered BM-MSCs into 11 patients with AIS grade A and found that five patients had their AIS grade improved to C [148]. Several studies have reported improvement in AIS grade in chronic SCI patients who received MSCs through the intraspinal route [149,150,151]. In contrast, Pal et al. reported that 30 BM-MSC-treated patients did not show any improvement or conversion in their AIS grades [152].

### 3.2. Umbilical Cord-Derived Mesenchymal Stem Cells

U-MSCs have several unique characteristics for use in SCI treatment, including ease of sourcing, excellent in vitro expansion, and fast proliferation. Furthermore, U-MSCs show low immunogenicity because they express very low or no expression of human leukocyte antigen typing, which is associated with immune rejection [128,153]. To avoid immune rejection, these cells utilize several additional mechanisms, including modulation of dendritic cell and T-cell functions and induction of regulatory T-cells [154].

U-MSCs have the ability to develop into a homogeneous population that expresses neural markers and develop neural phenotypic features [155]. These cells can be differentiated into multiple cell types, including neural-like and glial-like cells [156,157,158,159]. U-MSCs can be collected noninvasively, and their usage has not been hampered by ethical issues. They showed improved motor function and alleviated allodynia and hyperalgesia after SCI, via protection of neurons from apoptosis [160], inhibition of glial scar formation via regulation of metalloproteinase-2 [161], attenuation of ischemic damage of the spinal cord [162], and decreased reactive astrocytes in animal experiments [163,164,165,166].

Liu et al. found that 13 out of 22 patients showed improvement in the AIS quality of life in most patients with incomplete SCI [167]. In addition, Cheng et al. found that 7 out of 10 patients who received cell therapy demonstrated improvement in sensation, motion, muscle tension, and self-care ability [168]. Kang et al. reported improved motor function in the lower limb and expanded the atrophied spinal cord after injection of U-MSCs into the subarachnoid, intradural, or extradural space of the spinal cord in patients with compressed fractures [169].

### 3.3. Adipose-Derived Mesenchymal Stem Cells

Adipose tissue is distributed ubiquitously in the body and can be easily collected using minimally invasive techniques, such as liposuction or simple surgical interventions, and contains more somatic stem cells compared to bone marrow [170,171]. AD- and BM-MSCs share certain characteristics, such as cell morphology and expression of cell surface antigens. However, the rates of proliferation and multilineage capabilities markedly differ [172]. More somatic stem cells are contained in the adipose tissue than in bone marrow, which makes AD-MSCs a good MSC material for SCI treatment [170,171].

BM-MSCs are characterized by slow proliferation and higher osteogenic and chondrogenicity. In contrast, AD-MSCs exhibit higher proliferative activity and secrete higher levels of IGF-1, VEGF-D, and IL-8 [173]. Furthermore, the secretion levels of VEGF-A, angiogenin, bFGF, NGF, stem cell-derived factor-1, and HGF from BM-MSCs are comparable to those of AD-MSCs [174]. According to these findings, AD-MSCs tend to promote angiogenesis stimulation more strongly [174].

Intravenous AD-MSC administration improved hindlimb motor function via angiogenesis activation and up-regulation of extracellular signal-regulated kinase and Akt [175]. AD-MSCs also contribute to cell survival and tissue repair by increasing the expression of beta3-tubulin, BDNF, and ciliary neurotrophic factor [176]. The inflammatory response can also be down-regulated by the administration of AD-MSCs, which is mediated by blocking the infiltration of ED1 macrophages and attenuating Notch1 signaling [174,177,178]. In addition, the intrinsic ability of AD-MSCs to transdifferentiate into neuron/motoneuron-like cells may reduce cavitation and immune suppression by inhibiting astrocyte reactivation and the secretion of anti-inflammatory factors [179].

Ra et al. observed toxicity and tumorigenicity following intravenous injection of human AD-MSC in eight male patients with chronic SCI. There were no serious transplant-related adverse events in all patients during the 3-month follow-up period [180]. Hur et al. reported that 10 out of 14 SCI patients exhibited improvement in their sensory function, five patients experienced improvement of motor function, and two patients had improved voluntary anal contraction after administration of AD-MSCs [181]. Bydon et al. reported that treatment of an SCI patient with 100 million autologous AD-MSCs showed improvement in ASIA motor and sensory scores as well as improvement in the quality of life [182].

## 4. Why Should We Pay Attention to Extracellular Vesicles over Mesenchymal Stem Cells as a Therapeutic Source for Spinal Cord Injury?

MSCs have the potential to regenerate injured tissues or control the immunologic cascade; however, they also have significant limitations, particularly in view of carrying out clinical studies and developing therapeutic agents in real clinical practice. First, MSCs have a survival issue after cell implantation [183]. The longevity of MSCs may be driven by insufficient environment, cell niche, survival of MSCs, and poor intercellular communication between the cells. In particular, certain researchers have demonstrated the paradoxical period after implantation that pro-inflammatory activity surpasses anti-inflammatory activity in some phases [184]. Within this period, MSCs cannot survive sufficiently with reduced function. Second, MSCs are significantly heterogeneous due to the diversity of donor condition, type, differentiation capacity, and other factors between cells. In addition, MSCs are severely sensitive to the environment, resulting in negative effects on disease modulation, such as severe inflammation and active osteoarthritis [185,186]. Finally, the entire manufacturing process for MSCs, including ex vivo expansion, isolation technique, and cultivation method, has not yet been standardized; undetermined factors can affect the senescence and loss of capacity of implanted MSCs. To overcome these limitations of MSCs, extracellular vesicles (EVs), also called exosomes, have recently emerged as a novel source in the field of regenerative and anti-inflammatory medicine. Hence, as an alternative material in regenerative medicine, EVs should be considered for further research and development.

## 5. Overview and Characteristics of Extracellular Vesicles

**Figure 2:**
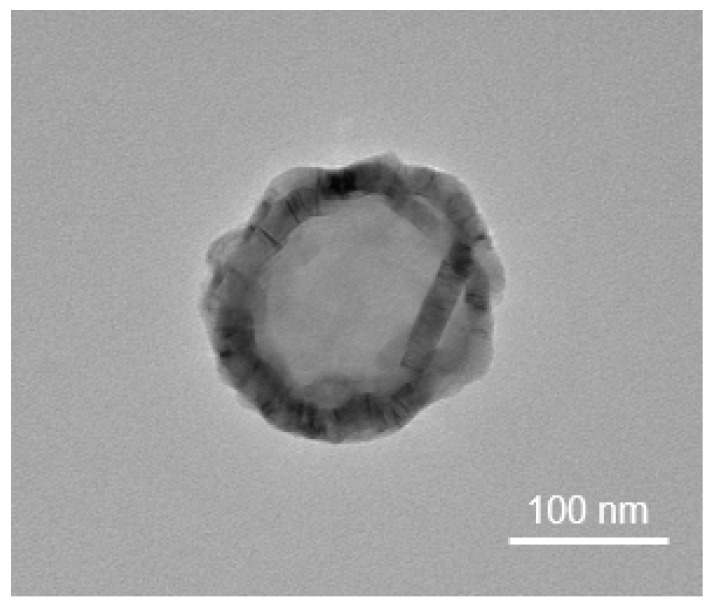
Transmission electron microscopy (TEM) image of MSC-derived EV morphology.

EVs are lipid bilayer vesicles derived from cells, serum, or other biological fluids (Figure 2). These are involved in biological signal transduction between cells and are emerging as mediators of disease therapeutics, diagnostic biomarkers, and drug delivery systems because of their ability to regulate various biological processes [187,188,189]. EVs are cell-derived vesicles that include cell-derived genetic materials and possess biological functional activity. EV cargo consists of bioactive molecules, including mRNAs, miRNAs, DNA, lipids, proteins, and metabolites. There are four types of EVs, categorized by their size and composition. Among them, exosomes are 50–200 nm in diameter and have a cup-shaped lipid bilayer membrane structure. Exosome membranes are enriched in cholesterol, ceramide, and sphingolipids, which are secreted through the budding of intraluminal vesicles and multivesicular bodies (MVBs) [190,191]. Exosomes are released by fusing MVBs with the cell membrane. However, another extracellular vesicle, microvesicles, is released from the cell through the outer bud of the cell membrane or apoptotic cell membrane [192].

Stem cell therapy and cell transplantation have been extensively studied and used as cell therapies in human clinical trials. In particular, MSCs are commonly used as cell-based therapeutics owing to their regenerative and immunosuppressive effects [193]. Currently, more than 600 clinical trials using MSCs are available at www.clinicaltrials.gov (5 November 2021) [194]. According to clinical trial information, MSCs are used to treat SCI, osteoarthritis, knee cartilage damage, and cancer. Damaged tissue regeneration is possible through the paracrine action of MSCs, and MSCs have been shown to be effective in healing and regenerating damaged tissues. However, there are certain disadvantages of systemically administered MSCs, including that they remain in the tissue for a long time to cause an immune response and are expensive and difficult to store as cell therapy, which need to be overcome. In recent years, MSC-based therapies have undergone multiple paradigm shifts to address these issues [194]. MSC-derived EVs are important because they show biological changes and regenerative effects as therapeutic agents for diseases and are safe as cell-free therapeutic agents. Currently, a clinical approach is underway to utilize MSC-derived EVs as a treatment for bone loss, diabetes mellitus type 1, Alzheimer’s disease, and sepsis (http://clinicaltrials.gov, accessed on 5 November 2021).

In contrast, the genetic material contained in EVs can be analyzed and used as a biomarker for disease diagnosis. miRNAs are packaged inside exosomes, and miRNAs from these secreted exosomes can be ideal biomarkers because of their high stability and degradation resistance. miRNAs are non-coding RNAs of 21–25 nucleotides, which regulate cellular responses after mRNA transcription [195]. Disease-specific miRNAs can be identified by comparing and analyzing the expression patterns of exosomal miRNAs from healthy individuals and exosomal miRNAs from patients. Circulating miRNAs are biomarkers for neurodegenerative diseases or cancers and can diagnose diseases early and observe prognosis [196,197]. For example, the let-7 miRNA family is abundant in the CNS and is involved in neurogenesis and increased cerebrospinal fluid in patients with Alzheimer’s disease [198,199].

The EVs use is one of the potentially promising approaches for delivering therapeutic agents to the CNS. EVs are miniscule and lipophilic vesicles that can be used in regenerative medicine, or to deliver drugs for anti-cancer, anti-inflammatory, and immune modulation across the blood-brain barrier (BBB) [200]. Effective treatment has become possible by modifying the surface of EVs or loading and delivering therapeutic substances to EVs. In addition, studies have reported the use of EVs as a drug delivery system to deliver therapeutic agents such as amyloid, miRNA-124, miRNA-133, siRNA, paclitaxel, and doxorubicin [201,202,203,204,205].

## 6. Research Trials Using Extracellular Vesicles for Spinal Cord Injury

To date, certain studies have evaluated the therapeutic efficacy of EVs for SCI in animal models. Numerous studies have demonstrated that EVs can pass through the BBB, reach the target lesion of the injured spinal cord, and positively affect the lesion. Guo et al. showed that intranasal EVs therapy could partly improve structural and electrophysiological function and, most importantly, significantly elicit functional recovery in rats with complete cord injury [206]. Kim HY et al. also demonstrated that accumulated EV-like nanovesicles enhanced blood vessel formation, attenuated inflammation and apoptosis in the injured spinal cord and consequently improved spinal cord function [207]. In addition, Zhong D, et al. reported that EVs could enhance the angiogenic activities in the injured spinal cord, with sufficient VEGF-A in the EVs, accelerated microvascular regeneration, reduced spinal cord cavity formation, and improved functional scores using the Basso mouse scale [208]. Together, these findings suggest that EVs possess many affirmative factors for the regeneration of injured spinal cords and, in the near future, may be a novel therapeutic agent for SCI in humans. However, in the literature and clinical trial registries, there have been no reports of clinical trials with EVs for SCI. For clinical trials, certain issues of the EVs, such as optimal reference for manufacturing and large quantity production, have to be proven entirely.

## 7. Conclusions and Future Perspectives

Functional recovery after SCI is considerably limited because of the very low plasticity and weak regenerative capacity of the CNS. Moreover, owing to the complex and long-term pathological process of SCI, recovery of the injured spinal cord is hampered by various factors. To date, SCI treatment is an unresolved challenge and there remains no effective strategy to restore the lost functions.

The various direct and indirect pathways for the regeneration of injured tissues possessed by MSCs and MSC-derived EVs have promising potential for SCI treatment. A series of small-scale patient trials revealed that MSC transplantation yielded better outcomes than the traditional treatments such as rehabilitation, including improvements in movement, sensation, and quality of life. Recently, MSC-derived EVs have become very popular in the field of regenerative medicine because they have various abilities to repair damaged tissues. Thus, MSC-derived EVs can be a good alternative material to overcome the inherent limitations of MSCs.

Certainly, various issues related to MSC-derived EVs, such as tissue sources, isolation, purification, and amplification, must be addressed first. To generate MSC-derived EVs in high yield and purity without affecting the biological activity of exosomes, it is necessary to establish a fast, inexpensive, and simple standardized isolation technique and purification procedure. In addition, along with the production of MSC-derived EVs, future trials to establish clinical effectiveness and safety in human applications should be conducted. Future studies to establish a comprehensive theoretical basis for the clinical application of MSC-derived EVs will provide a direction and hope for the clinical treatment of SCI.

## Data Availability

Data sharing not applicable.
